# The Influencing Factors of Physical Exercise on the Social and Emotional Abilities of College Students: A Chain‐Mediated Effect Based on Emotion Regulation and Self‐Efficacy

**DOI:** 10.1155/da/8879310

**Published:** 2026-04-01

**Authors:** Longan Cao, Jianhui Liu, Lei Wang, Jun Lang, Zhengban Ran

**Affiliations:** ^1^ School of Physical Education, Southwest University, Chongqing, China, southwest.edu; ^2^ School of Physical Education, Jiangxi Science and Technology Normal University, Jiangxi, China, jxstnu.cn; ^3^ Teaching and Research Association, Guang’an Institute of Technology, Sichuan, China

**Keywords:** chain intermediation, college student, physical exercise, social–emotional competence

## Abstract

The university period represents a critical transition for college students, marking their progression from adolescence to adulthood and from the familial and school environments into broader society. Focusing on the enhancement of students’ social–emotional competence aligns with the developmental requirements of educational modernization in China. However, under the combined influence of severe external environments and internal factors, an increasing number of university students are prone to developing negative emotions, which poses potential risks to the healthy development of school education and social interactions. This study investigates how physical exercise influences the social–emotional competence of university students at Jiangxi Science and Technology Normal University, focusing on the mediating roles of emotion regulation and self‐efficacy. Using literature review, questionnaires, and statistical analyses on a sample of 572 students, the study assessed physical activity levels, emotion regulation strategies, self‐efficacy, and social–emotional competence. Results show that students generally engage in moderate physical activity, though over half exhibit low activity levels, cognitive reappraisal is used more frequently than expressive suppression, while both self‐efficacy and social–emotional competence are above average but need improvement. Significant differences across demographic variables were found in physical exercise, emotion regulation, self‐efficacy, and social–emotional competence. All four variables demonstrated significant positive correlations. Regression and mediation analyses revealed that physical exercise positively predicts social–emotional competence directly and indirectly through emotion regulation and self‐efficacy, with both independent and serial mediation effects. These findings clarify the mechanism linking physical exercise to social–emotional competence and provide theoretical support for promoting students’ socioemotional development through enhanced physical activity.

## 1. Introduction

It is widely recognized in the international education community that an individual’s development, success, and well‐being result from the combined effects of cognitive abilities and social–emotional competencies. The latter represents a key skill set essential for addressing various challenges in the 21st century. The university period marks a critical transition for students, bridging adolescence and adulthood, and serving as a pivotal phase from family life to broader societal engagement. Focusing on the enhancement of students’ social–emotional competence aligns with the developmental demands of educational modernization in China. Therefore, how to improve university students’ social–emotional competence in higher education, facilitate the construction of their social identity, and establish promotion models that foster the development of such competence have become significant theoretical and practical issues that must be addressed to achieve balanced development in contemporary higher education [1]. However, amid an increasingly complex, diverse, and uncertain growth environment, social–emotional competence remains a persistent challenge for many students. In past educational practices, an excessive focus on score improvement, while conducive to cognitive development, has overlooked social–emotional competence as a core noncognitive element [2]. This has led to a decline in the overall efficiency of educational outcomes, considerably limiting graduates’ competitiveness in the labor market [3] and made young adults entering society highly susceptible to negative emotions such as depression. Against the backdrop of globalization and the information age, intensifying social competition and rising productivity demands require individuals to possess more comprehensive and holistic competencies [4].

In view of this, as the core entity upon which higher education depends, university students’ level of social–emotional competence not only reflects the quality of education in the new era but also fundamentally relates to how universities should approach the mission of fostering character and civic virtue [5]. Consequently, a new pivotal task for higher education research is exploring how to guide students in effectively utilizing campus resources and environments, and transforming these into active forces for developing their social–emotional competence [6].

Social–emotional competence, also referred to as noncognitive skills, soft skills, or emotional intelligence, was initially introduced by the Collaborative for Academic, Social, and Emotional Learning (CASEL) in 1994. According to CASEL’s framework, this competency encompasses multiple core skill sets, including self‐awareness and self‐management at the individual level, as well as social awareness and relationship skills at the interpersonal level, along with responsible decision‐making ability demonstrated in the process of decision‐making [7]. The Organisation for Economic Co‐operation and Development (OECD), drawing on the Big Five personality theory, defines social–emotional competence by emphasizing its manifestations in task performance, emotion regulation, collaborative ability, open‐mindedness, and engagement skills. Adopting an innovative relationship‐centered perspective, Du Yuan and Mao Yaqing systematically deconstructed this competence and developed a theoretical framework comprising six dimensions: self‐cognition and self‐management at the individual level, other‐cognition and other‐management at the interpersonal level, and collective cognition and collective management at the group level [8]. Therefore, this study defines social–emotional competence as a series of key skills closely related to social development that university students demonstrate when confronted with diverse and constantly changing complex life situations [9]. These skills span both cognitive and managerial dimensions, specifically categorized into six domains: the cognition and management of the self, others, and the collective.

The term “physical exercise” has a long history in China. It was defined in the 1984 Chinese publication Sports Dictionary as “a process of engaging in physical activities through various sports methods and means, combined with natural elements such as sunlight, aimed at improving health” [10]. International scholars often distinguish the concept of physical exercise from broader physical activity and physical fitness training, noting that physical exercise represents a planned, structured, and repetitive subset of physical activity conducted with the explicit objective of improving or maintaining physical health [11]. Based on embodied cognition theory—which posits that bodily states directly influence cognitive and physiological processes, as well as interactions with the external environment—physical exercise can be assumed to exert a direct impact on social–emotional competence. Through exercise, individuals optimize their physical functions, which positively affects cognition, thereby enabling them to master challenging tasks, surpass their prior standards, strive to demonstrate social–emotional competence, and ultimately achieve satisfactory outcomes. Consistent with previous findings, physical exercise not only promotes positive physical and emotional states but also reduces individuals’ physiological sensitivity to stress [12]. In turn, well‐developed social–emotional competence can further encourage participation in physical exercise, thereby contributing to the development and improvement of the cardiovascular and respiratory systems, reducing muscle tension, and enhancing students’ motor coordination. Thus, it can be established that physical exercise and social–emotional competence exhibit a mutually reinforcing and synergistic relationship. However, although schools are recognized as ideal settings for promoting physical exercise, enhancing achievement motivation, and fostering students’ social–emotional competence, and although research on increasing university students’ interest in physical exercise has grown significantly, there remains a notable lack of comprehensive data that integrate physical education cases within universities to outline holistic approaches for improving social–emotional competence. More critically, the development of university students’ social–emotional competence is coinfluenced by multiple factors such as family background, school environment, and place of residence, leading to corresponding disparities in its manifestation, without marked leapfrog improvements in their social–emotional outcomes. Therefore, this study focused on Chinese college students and listed: H1: physical exercise is positively related to social–emotional competence.

Although physical exercise may directly enhance university students’ social–emotional competence, current understanding of whether this process is influenced by other factors remains incomplete, necessitating further investigation. Emotion regulation refers to the process by which individuals manage and modulate their emotional experiences [13], playing a critical role in maintaining physiological and psychological well‐being. Among various emotion regulation strategies, cognitive reappraisal and expressive suppression, as proposed by Gross and John, are two widely examined strategies in empirical research [14]. Cognitive reappraisal, an antecedent‐focused strategy, occurs prior to the full activation of emotional response and involves reinterpreting the meaning of a situation to alter its emotional impact. Expressive suppression, in contrast, is a response‐focused strategy that occurs during the emotional response phase and entails the conscious inhibition of emotion‐expressive behavior. Research indicates that physical exercise can strengthen an individual’s psychological architecture, thereby supporting their capacity to cope with life difficulties [15]. Furthermore, emotion regulation moderates individuals’ social–emotional competence, as positive social emotions contribute to its refinement [16]. Therefore, physical exercise may positively influence emotion regulation, which in turn may enhance students’ social–emotional competence. Additionally, given that emotion regulation constitutes an active process spanning multiple stages of emotional response, with different strategies exerting distinct effects on emotion generation and expression, this study will examine both cognitive reappraisal and expressive suppression separately to provide a more comprehensive assessment of individual differences in emotion regulation characteristics and efficacy. Accordingly, the following hypothesis is proposed: H2: Emotion regulation, specifically both cognitive reappraisal and expressive suppression strategies, mediates the relationship between physical exercise and university students’ social–emotional competence.

In both academic and broader social contexts, university students inevitably encounter setbacks and difficulties that may evoke tendencies toward withdrawal. Therefore, enhancing their social–emotional competence is imperative. Within this framework, self‐efficacy is defined as an individual’s perception or belief in their capability to effectively manage various aspects of their social lives [17]. As one of the psychological variables most closely associated with physical exercise behavior, scholars posit that self‐efficacy plays a significant mediating role in the relationship between university students’ physical exercise and their social–emotional competence. According to self‐efficacy theory, on the one hand, positive physical and mental states help individuals build self‐efficacy. Thus, physical exercise may enhance self‐efficacy by fostering social–emotional competence—for instance, students who regularly engage in physical exercise tend to exhibit higher levels of self‐efficacy. On the other hand, self‐efficacy influences individuals’ thought patterns, emotional responses, and affective experiences. Those with high self‐efficacy are more likely to adopt appropriate strategies in the face of failure or academic pressure, thereby resisting stress and negative emotions and demonstrating stronger social–emotional competence [18]. It is, thus, plausible that self‐efficacy is influenced by physical exercise and, in turn, affects students’ social–emotional competence. Based on this reasoning, the following hypothesis is proposed: H3: Self‐efficacy mediates the relationship between physical exercise and university students’ social–emotional competence.

Although existing literature indicates that emotion regulation and self‐efficacy independently influence the relationship between physical exercise and social–emotional competence, these factors may also operate sequentially, forming an innovative and coherent explanatory framework. For instance, students with strong emotion regulation abilities can effectively resolve psychological issues, thereby enhancing their self‐efficacy. Furthermore, self‐efficacy is influenced by factors such as direct and indirect experiences, physical and mental states, and cognitive appraisal, while also acting through multiple mechanisms to affect an individual’s cognition, behavior, and emotional responses. This dynamic is manifested in the ongoing interaction between the individual and their environment, ultimately contributing to a higher level of social–emotional competence that helps mitigate the risks of both externalizing and internalizing problems. In summary, there is reasonable ground to argue that physical exercise can provide individuals with positive emotional experiences, enable effective management of emotion regulation, and thereby foster positive self‐efficacy, which, in turn, mobilizes more adaptive social–emotional competence and promotes healthier interpersonal functioning. As articulated in the following: H4: Emotion regulation and self‐efficacy play a serial mediating role in the relationship between physical exercise and university students’ social–emotional competence.

Demographic variables (gender, grade level, major, and place of residence) have consistently received significant attention in previous research on social–emotional competence. While most existing studies have primarily focused on adolescents or community‐dwelling older adults, the present study specifically concentrates on university students as the target population.

In sum, this study establishes physical exercise among university students as the independent variable, social–emotional competence as the dependent variable, and emotion regulation and self‐efficacy as mediating variables, constructing a hypothesized mediation model. Within this framework, we examine the mechanisms through which physical exercise influences social–emotional competence, ultimately aiming to address gaps in long‐term and multifaceted programs aimed at enhancing this competence. In this regard, we seek to provide university administrators with theoretical management strategies for strengthening students’ social–emotional capacities, while also offering practical insights regarding physical activity for improving future professional interpersonal interactions among university students.

## 2. Materials and Methods

### 2.1. Participants and Procedure

The survey employed a convenience sampling method, selecting 600 university students from Jiangxi Science and Technology Normal University as participants. Prior to the study, university administrators were contacted, and informed consent was obtained from all student participants. Questionnaires were distributed to first‐ and second‐year students through their general physical education instructors on a class‐by‐class basis, and completed questionnaires were collected immediately. For third‐ and fourth‐year students, a random sampling approach was adopted. A total of 600 questionnaires were distributed. After excluding responses with completion times of less than 10 min, missing answers, and invalid submissions, 572 valid questionnaires were retained, resulting in a response rate of 95.33%. The valid sample included 154 first‐year students, 159 second‐year students, 123 third‐year students, and 136 fourth‐year students. The sample distribution is presented in Table 1.

**Table 1:** Demographic characteristics.

Variable	Level	*N*	Percentage (%)	Cumulative percentage (%)
Gender	Male	259	45.28	45.28
	Female	313	54.72	100

Grade	Freshmen	154	26.92	26.92
	Sophomores	159	27.8	54.72
	Juniors	123	21.5	76.22
	Seniors	136	23.78	100

Degree subject	Social sciences major	239	41.78	41.78
	Natural sciences major	333	58.22	100

Place of residence	City	321	56.12	56.12
	Villages	251	43.88	100

Physical exercise grade	Low exercise level	291	50.87	50.87
	Moderate exercise level	108	18.88	69.76
	High exercise level	173	30.24	100

According to national laws and institutional regulations, the collection of survey data has obtained written informed consent from all participants. Upon completion of the survey, the research team provided each participant with a small token of appreciation (a commemorative gift valued at 2 RMB) in recognition of their time and contribution.

### 2.2. Materials

#### 2.2.1. Physical Exercise

The study utilized the Physical Activity Rating Scale (PARS‐3), revised by Liang [19], to assess the physical exercise level of university students. This scale has been widely used among Chinese university student populations, demonstrating sound applicability and empirical support. PARS‐3 measures physical exercise across three dimensions: exercise intensity (A), duration per session (B), and exercise frequency (C). Each dimension is assessed by a single item rated on a 1–5 scale. The total physical exercise score is calculated as follows: intensity × (duration − 1) × frequency, yielding a score ranging from 0 to 100. Based on this score, participants are categorized into three exercise levels: low (≤19 points), moderate (20–42 points), and high (≥43 points). The scale has shown good reliability and validity and was used in its original form in this study. The Cronbach’s *α* coefficient in the present study was 0.84.

#### 2.2.2. University Students Social–Emotional Competence Scale

Social–emotional competence was measured using the Social–Emotional Competence Scale, developed under the “Social–Emotional Learning” project jointly initiated by the Ministry of Education of China and UNICEF, and it was gradually improved by the revision of Chinese scholar [20]. The scale comprises six dimensions: self‐cognition, others‐cognition, collective‐cognition, self‐management, others‐management, and collective‐management, with a total of 61 items. Responses were recorded on a 5‐point Likert scale ranging from 1 (“completely inconsistent”) to 5 (“completely consistent”). Higher total scores indicate a higher level of social–emotional competence among university students. The scale has demonstrated good reliability and validity, with a Cronbach’s *α* coefficient of 0.92 in this study. Details are shown in Table 2.

**Table 2:** List of reliability tests for the social–emotional strength scale.

Dimensionality	Question title	Corresponding number	Cronbach’s alpha
Autognosis	8	1–8	0.91
Intersubjectivity	7	9–15	0.89
Collective cognition	5	16–20	0.875
Self‐management	17	21–37	0.952
Other management	11	38–48	0.934
Collective stewardship	13	49–61	0.942

#### 2.2.3. Emotion Regulation Questionnaire (ERQ)

The Gross–John ERQ, originally developed by Gross and John [13] was used to assess emotion regulation strategies. In previous studies, the Cronbach’s *α* coefficients for the cognitive reappraisal and expressive suppression subscales were 0.85 and 0.77, respectively. The scale consists of 10 items covering two dimensions: cognitive reappraisal and expressive suppression. Items are rated on a 7‐point Likert scale (1 = strongly disagree and 7 = strongly agree). Higher scores on each subscale indicate a greater tendency to use the corresponding emotion regulation strategy. The items corresponding to each dimension are listed in Table 3.

**Table 3:** List of reliability tests for the emotional regulation scale.

Dimensionality	Question title	Corresponding number	Cronbach’s alpha
Cognitive reappraisal	6	1, 3, 5, 7, 8, 10	0.94
Expressive suppression	4	2, 4, 6, 9	0.90

#### 2.2.4. General Self‐Efficacy Scale (GSES)

The Chinese version of the GSES was used to assess individuals’ levels of self‐efficacy [21], which represents their subjective evaluation of confidence in performing specific behaviors (e.g., “I can always solve problems if I try hard enough.”) The scale consists of 10 items and follows a unidimensional structure. Each item is rated on a 4‐point Likert scale, ranging from “completely inconsistent” (1 point) to “completely consistent” (4 points). The total score ranges from 10 to 40, with higher scores indicating stronger self‐efficacy. The GSES has been widely used among Chinese university student populations and has demonstrated good reliability and validity [22]. In this study, the scale exhibited good internal consistency, with a Cronbach’s *α* coefficient of 0.93.

### 2.3. Data Analysis

Statistical analysis is one of the core research methods in this study, which plays a pivotal role. It encompassed systematic data organization and in‐depth analysis. Data were analyzed using SPSS 27.0 and the SPSS plugin Process 3.5. (All the original data are presented in the Supporting Information.) The analytical procedure was conducted as follows: First, tests for normality and common method bias were performed on the sample data. Second, descriptive statistics were computed, presenting means and standard deviations for all variables. Third, independent samples *t*‐tests and one‐way analysis of variance (ANOVA) were employed to examine differences across demographic variables [23]. Fourth, Pearson’s bivariate correlation analysis and linear regression analysis were conducted to examine the relationships among all variables. Finally, Model 6 of the SPSS Process 3.5 macro was utilized to delve into the predictive effect of physical exercise on university students’ social–emotional competence and to test the hypothesized serial mediation model involving emotion regulation (cognitive reappraisal and expressive suppression) and self‐efficacy. A bootstrap sampling method with 5000 resamples was applied to derive robust standard errors and 95% bias‐corrected confidence intervals (CIs). A significant effect is indicated if the CI does not contain zero [24].

## 3. Results

### 3.1. Common Method Bias Test

All data in this study were collected via self‐report questionnaires, which may introduce common method bias, potentially confounding the results. To ensure the accuracy of the questionnaire findings, Harman’s single‐factor test was conducted to assess common method bias. The results indicated that among the 10 factors extracted, the maximum factor explained 29.73% of the variance, which is below the critical threshold of 40% [22]. Thus, common method bias was not considered a substantial concern in this study. The normality of the sample data was evaluated using kurtosis and skewness indices. As shown in Table 4, the absolute values of kurtosis for all variables (physical exercise, emotion regulation, self‐efficacy, and social–emotional competence, along with their subfactors) were below 7, and the absolute values of skewness were below 2 [23]. Accordingly, the data were considered to approximate a normal distribution.

**Table 4:** Descriptive statistical analysis summary table.

Variables	Dimension	*M*	SD	Min	Max	Kurtosis	Skewness
Physical exercise		29.477	27.682	0	100	−0.283	0.863
	Activity intensity	3.103	1.257	1	5	−0.988	−0.047
	Activity time	3.182	1.225	1	5	−0.925	−0.127
	Motion frequency	3.178	1.206	1	5	−0.853	−0.112
Emotion regulation		41.483	16.023	13	69	−1.139	0.224
	Cognitive reappraisal	24.792	10.47	7	42	−1.374	0.233
	Expressive suppression	16.691	6.864	4	28	−1.336	0.157
Self‐efficacy		28.752	7.569	10	40	−0.476	−0.332
Social–emotional competence		203.911	38.164	82	279	0.621	−0.796
	Autognosis	26.696	7.123	10	40	−0.853	−0.024
	Intersubjectivity	23.505	6.131	9	35	−0.847	−0.127
	Collective cognition	16.734	4.657	5	25	−0.779	−0.148
	Self‐management	56.556	14.295	22	85	−0.833	−0.018
	Other management	36.818	9.718	13	55	−0.837	−0.069
	Collective stewardship	43.601	11.311	14	65	−0.862	−0.085

### 3.2. Descriptive Analysis

Based on the descriptive statistical analysis of the four key indicators—physical exercise, emotion regulation, self‐efficacy, and social–emotional competence—along with their respective subdimensions, the specific characteristics of the surveyed college student population are summarized as follows: As shown in Table 4, the total physical activity score of the participants was 29.48 ± 27.68. According to the grading criteria established by the Physical Activity Rating Scale, this result indicates that the average physical exercise level of this group falls within the moderate range. In terms of emotion regulation, the scores for cognitive reappraisal and expressive suppression were 24.79 ± 10.47 and 16.69 ± 6.84, respectively. This suggests that the frequency of using cognitive reappraisal strategies is generally higher than that of expressive suppression among these students. Regarding self‐efficacy, the average score was 28.75 ± 7.57, with a minimum value of 10 and a maximum of 40. According to the measurement standard of this scale, higher scores reflect stronger self‐efficacy. For social–emotional competence, the mean score was 203.91 ± 38.16, ranging from 82 to 279. Similarly, higher scores on this scale indicate a stronger overall level of social–emotional competence among the students.

### 3.3. Analysis of Group Differences

As shown in the Table 5, no significant differences were observed in the emotion regulation dimension among college students of different genders. However, significant differences were identified in the three dimensions of physical exercise, self‐efficacy, and social–emotional competence.

**Table 5:** Differences analysis table.

Variables	Physical exercise	Emotion regulation	Self‐efficacy	Social–emotional competence
Gender				
Male (*n* = 259)	32.93 ± 30.62	42.84 ± 15.49	29.83 ± 7.71	209.16 ± 35.56
Female (*n* = 313)	26.62 ± 24.68	40.36 ± 16.39	27.86 ± 7.34	199.57 ± 39.73
*t*	2.672	1.844	3.123	3.012
*p*	0.008 ^∗∗^	0.066	0.002 ^∗∗^	0.003 ^∗∗^
Grade				
Freshmen (*n* = 154)	25.18 ± 22.93	41.14 ± 16.39	27.81 ± 7.81	200.23 ± 43.88
Sophomores (*n* = 159)	28.87 ± 25.62	41.99 ± 15.78	28.16 ± 6.79	201.35 ± 34.84
Juniors (*n* = 123)	28.11 ± 26.52	39.85 ± 15.64	29.76 ± 7.70	202.32 ± 36.33
Seniors (*n* = 136)	36.28 ± 34.23	42.75 ± 16.26	29.60 ± 7.90	212.51 ± 35.57
*F*	4.166	0.784	2.436	3.121
*p*	0.006 ^∗∗^	0.503	0.064	0.026 ^∗^
Degree subject				
Social sciences major (*n* = 239)	33.31 ± 31.18	41.74 ± 15.42	29.21 ± 7.67	210.71 ± 31.19
Natural sciences major (*n* = 333)	26.73 ± 24.55	41.30 ± 16.47	28.42 ± 7.49	199.03 ± 41.84
*t*	2.712	0.321	1.225	3.822
*p*	0.007 ^∗∗^	0.748	0.221	0.000 ^∗∗^
Place of residence				
City (*n* = 321)	32.55 ± 29.28	42.17 ± 15.51	29.27 ± 7.94	208.38 ± 34.43
Villages (*n* = 251)	25.55 ± 25.01	40.60 ± 16.65	28.08 ± 7.03	198.20 ± 41.85
*t*	3.077	1.168	1.871	3.116
*p*	0.002 ^∗∗^	0.243	0.062	0.002 ^∗∗^

^∗^ *p* < 0.05.

^∗∗^ *p* < 0.01.

Regarding grade level, no significant differences (*p*  > 0.05) were found in self‐efficacy and emotion regulation among students of different grades, indicating relative consistency in these dimensions across grade levels. In contrast, significant differences (*p*  < 0.05) were observed in physical exercise and social–emotional competence, suggesting variations in physical activity frequency, interpersonal management, and social–emotional abilities among students from different grades.

For major and place of origin, no significant differences (*p*  > 0.05) were detected in emotion regulation and self‐efficacy. However, significant differences (*p*  < 0.05) were present in physical exercise and social–emotional competence.

### 3.4. Correlation Analysis

Pearson’s two‐tailed correlation analysis was conducted to examine the relationships among university students’ physical exercise, social–emotional competence, emotion regulation, and self‐efficacy. As shown in Table 6, all variables in the sample demonstrated significant pairwise correlations (*p*  < 0.01). Specifically, physical exercise showed moderate correlations (0.3 < |*r*| < 0.5) with self‐efficacy, self‐cognition, others‐cognition, collective‐cognition, self‐management, others‐management, and collective‐management, while exhibiting strong correlations (|*r*| > 0.5) with emotion regulation, cognitive reappraisal, expressive suppression, and social–emotional competence. Emotion regulation was moderately correlated with self‐efficacy and all social–emotional competence subdimensions (self‐cognition, others‐cognition, collective‐cognition, self‐management, others‐management, and collective‐management), and strongly correlated with cognitive reappraisal, expressive suppression, and overall social–emotional competence. Self‐efficacy demonstrated moderate correlations with all subdimensions of social–emotional competence, cognitive reappraisal, and expressive suppression but was strongly correlated with overall social–emotional competence. Social–emotional competence was strongly correlated with each of its subdimensions (|*r*| > 0.5). These results support further regression or mediation model testing.

**Table 6:** Correlation analysis summary table.

Variables	*M*	SD	1	2	3	4	5	6	7	8	9	10	11	12
Physical exercise	29.48	27.68	1	—	—	—	—	—	—	—	—	—	—	—
Self‐efficacy	28.75	7.57	0.48 ^∗∗^	1	—	—	—	—	—	—	—	—	—	—
Autognosis	26.70	7.12	0.39 ^∗∗^	0.32 ^∗∗^	1	—	—	—	—	—	—	—	—	—
Intersubjectivity	23.51	6.13	0.42 ^∗∗^	0.33 ^∗∗^	0.44 ^∗∗^	1	—	—	—	—	—	—	—	—
Collective cognition	16.73	4.66	0.38 ^∗∗^	0.36 ^∗∗^	0.36 ^∗∗^	0.38 ^∗∗^	1	—	—	—	—	—	—	—
Self‐management	56.56	14.29	0.47 ^∗∗^	0.39 ^∗∗^	0.40 ^∗∗^	0.45 ^∗∗^	0.40 ^∗∗^	1	—	—	—	—	—	—
Other management	36.82	9.72	0.45 ^∗∗^	0.39 ^∗∗^	0.40 ^∗∗^	0.40 ^∗∗^	0.42 ^∗∗^	0.47 ^∗∗^	1	—	—	—	—	—
Collective stewardship	43.60	11.31	0.43 ^∗∗^	0.40 ^∗∗^	0.37 ^∗∗^	0.39 ^∗∗^	0.40 ^∗∗^	0.35 ^∗∗^	0.38 ^∗∗^	1	—	—	—	—
Cognitive reappraisal	24.79	10.47	0.70 ^∗∗^	0.47 ^∗∗^	0.39 ^∗∗^	0.36 ^∗∗^	0.35 ^∗∗^	0.40 ^∗∗^	0.42 ^∗∗^	0.39 ^∗∗^	1	—	—	—
Expressive suppression	16.69	6.86	0.64 ^∗∗^	0.45 ^∗∗^	0.39 ^∗∗^	0.40 ^∗∗^	0.36 ^∗∗^	0.40 ^∗∗^	0.43 ^∗∗^	0.41 ^∗∗^	0.70 ^∗∗^	1	—	—
Social–emotional competence	203.91	38.16	0.60 ^∗∗^	0.52 ^∗∗^	0.66 ^∗∗^	0.68 ^∗∗^	0.62 ^∗∗^	0.79 ^∗∗^	0.73 ^∗∗^	0.70 ^∗∗^	0.55 ^∗∗^	0.56 ^∗∗^	1	—
Emotion regulation	41.48	16.02	0.73 ^∗∗^	0.50 ^∗∗^	0.42 ^∗∗^	0.40 ^∗∗^	0.39 ^∗∗^	0.44 ^∗∗^	0.46 ^∗∗^	0.43 ^∗∗^	0.95 ^∗∗^	0.88 ^∗∗^	0.60 ^∗∗^	1

^∗∗^ *p* < 0.01.

### 3.5. Collinearity Diagnostics

The collinearity diagnostics for physical exercise, emotion regulation, and self‐efficacy among university students (Table 7) indicated that all variables had condition indices below 30, variance inflation factors (VIFs) below 5, and tolerance values above 0.3. Thus, no multicollinearity issues were detected, supporting the feasibility of subsequent analyses.

**Table 7:** List of diagnostic criteria for collinearity.

Variables	VIF	Tolerance values
Physical exercise	1.677	0.596
Self‐efficacy	1.364	0.733
Emotion regulation	1.84	0.544

### 3.6. Regression Analysis of Physical Exercise, Emotion Regulation, Self‐Efficacy, and Social–Emotional Competence

As shown in Table 8, the overall regression model was statistically significant (*R* ^2^ = 0.458, *F* = 59.5, *p*  < 0.01). The regression analysis indicated that physical exercise significantly and positively predicted university students’ social–emotional competence (*β* = 0.469, *p*  < 0.01). Furthermore, physical exercise significantly predicted both cognitive reappraisal (*β* = 0.614, *p*  < 0.01) and expressive suppression (*β* = 0.546, *p*  < 0.01).

**Table 8:** Regression analysis overview of social–emotional competence on physical exercise, emotional regulation, and self‐efficacy(*N* = 572).

Outcome variable	Predictor variable	*R* ^2^	Adjust *R* ^2^	*F*	*β*	*T*
Social–emotional competence	Gender	0.259	0.253	39.624	−0.063	−1.714
	Grade				0.016	0.423
	Degree subject				−0.077	−2.049 ^∗^
	Place of residence				−0.049	−1.291
	Physical exercise				0.469	12.693 ^∗∗^

Cognitive reappraisal	Gender	0.366	0.361	65.44	0	0.004
	Grade				−0.07	−1.993 ^∗^
	Degree subject				0.044	1.251
	Place of residence				0.014	0.395
	Physical exercise				0.614	17.968 ^∗∗^

Expressive suppression	Gender	0.298	0.291	47.939	−0.036	−1.021
	Grade				−0.022	−0.612
	Degree subject				0.045	1.22
	Place of residence				0.004	0.1
	Physical exercise				0.546	15.181 ^∗∗^

Self‐efficacy	Gender	0.279	0.27	31.122	−0.071	−1.96
	Grade				0.059	1.583
	Degree subject				−0.006	−0.158
	Place of residence				−0.024	−0.636
	Physical exercise				0.153	3.266 ^∗∗^
	Cognitive reappraisal				0.23	4.274 ^∗∗^
	Expressive suppression				0.199	3.899 ^∗∗^

Social–emotional competence	Gender	0.458	0.45	59.5	−0.033	−1.054
	Grade				0.023	0.72
	Degree subject				−0.1	−3.057 ^∗∗^
	Place of residence				−0.047	−1.447
	Physical exercise				0.122	2.966 ^∗∗^
	Cognitive reappraisal				0.172	3.635 ^∗∗^
	Expressive suppression				0.25	5.557 ^∗∗^
	Self‐efficacy				0.261	7.145 ^∗∗^

^∗^ *p* < 0.05.

^∗∗^ *p* < 0.01.

When physical exercise and emotion regulation were included together to predict self‐efficacy and physical exercise positively predicted self‐efficacy (*β* = 0.153, *p*  < 0.01). Additionally, cognitive reappraisal significantly predicted self‐efficacy (*β* = 0.230, *p*  < 0.01), as did expressive suppression (*β* = 0.199, *p*  < 0.01).

When physical exercise, emotion regulation (both subdimensions), and self‐efficacy were included simultaneously to predict social–emotional competence, all four predictors demonstrated significant effects: physical exercise (*β* = 0.122, *p*  < 0.01), cognitive reappraisal (*β* = 0.172, *p*  < 0.01), expressive suppression (*β* = 0.250, *p*  < 0.01), and self‐efficacy (*β* = 0.261, *p*  < 0.01).

### 3.7. Mediation Effect Test: The Impact of Physical Exercise on Social–Emotional Competence

Based on the proposed hypotheses, a serial mediation model was tested with physical exercise as the independent variable, social–emotional competence as the dependent variable, and emotion regulation (including its subdimensions: cognitive reappraisal and expressive suppression) and self‐efficacy as mediators. Model 6 of the SPSS Process 3.5 macro was employed, using a bootstrap approach with 5000 resamples to examine the serial mediation effects. The results are presented in Table 9.

**Table 9:** Summary table of bootstrap analysis for the mediation effect test of emotion regulation and self‐efficacy.

Path	Total					Positive coping style					Negative coping style				
	Effect size	SE	95% CI		Proportion (%)	Effect size	SE	95% CI		Proportion (%)	Effect size	SE	95% CI		Proportion (%)
			Boot LLCL	Boot ULCL				Boot LLCL	Boot ULCL				Boot LLCL	Boot ULCL	
Total mediating effect	0.68	0.05	0.582	0.779	100	0.68	0.05	0.582	0.779	100	0.68	0.05	0.582	0.779	100
Direct effect	0.269	0.055	0.162	0.377	39.56	0.268	0.056	0.157	0.379	39.41	0.271	0.053	0.166	0.375	39.85
Total indirect effect	0.411	0.055	0.309	0.519	60.44	0.413	0.056	0.307	0.525	60.59	0.41	0.053	0.311	0.514	60.15
Indirect 1	0.239	0.037	0.167	0.313	35.15	0.236	0.039	0.161	0.313	34.62	0.241	0.035	0.174	0.313	35.36
Indirect 2	0.096	0.027	0.049	0.155	14.11	0.092	0.027	0.046	0.153	13.5	0.099	0.027	0.053	0.158	14.52
Indirect 3	0.076	0.016	0.045	0.113	11.18	0.085	0.018	0.052	0.121	12.47	0.07	0.015	0.043	0.102	10.27
C1: Ind1–Ind2	0.143	0.043	0.057	0.226	—	0.144	0.044	0.056	0.229	—	0.143	0.042	0.058	0.223	—
C2: Ind1–Ind3	0.162	0.037	0.088	0.244	—	0.152	0.040	0.073	0.232	—	0.171	0.035	0.103	0.245	—
C3: Ind2–Ind3	0.018	0.031	−0.036	0.086	—	0.007	0.032	−0.051	0.077	—	0.029	0.028	−0.02	0.092	—

In both, the overall sample and subsamples grouped by different regulation strategies, physical exercise not only exerted a significant direct effect on social–emotional competence but also showed significant indirect effects through the following pathways: Indirect Path 1: physical exercise → emotion regulation (total/cognitive reappraisal/expressive suppression) → social–emotional competence. Indirect Path 2: physical exercise → self‐efficacy → social–emotional competence. Indirect Path 3: physical exercise → emotion regulation (total/cognitive reappraisal/expressive suppression) → self‐efficacy → social–emotional competence.

**Figure 1:**
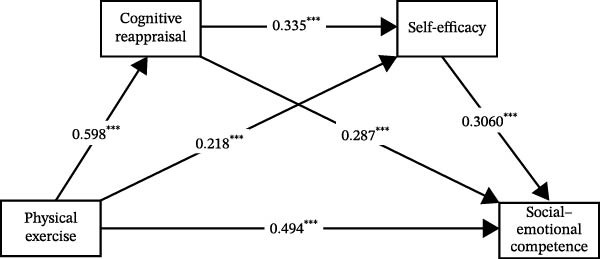
The chain mediation effect path diagram of cognitive reappraisal and self‐efficacy.  ^∗∗∗^ *p* < 0.001.

**Figure 2:**
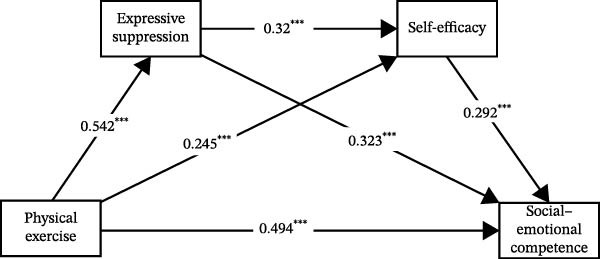
The chain mediation effect path diagram of expressive suppression and self‐efficacy.  ^∗∗∗^ *p* < 0.001.

**Figure 3:**
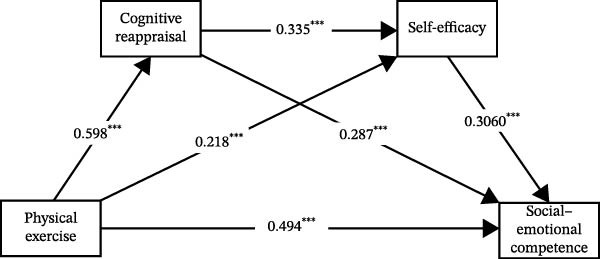
The chain mediation effect path diagram of emotion regulation and self‐efficacy.  ^∗∗∗^ *p* < 0.001.

In summary, based on the integrated results above, the final mediation model with all significant pathways was derived and is illustrated in Figures 1–3.

## 4. Discussion

This study investigated the predictive mechanism of physical exercise on university students’ social–emotional competence, constructing a mediation model with emotion regulation and self‐efficacy as mediating factors.

First, this research confirms that physical exercise serves as a significant predictor of university students’ social–emotional competence. The findings demonstrate that physical exercise has a direct and positive predictive effect on social–emotional competence, indicating that engaging in physical exercise can enhance this capability, which aligns with existing research findings [25]. The work of scholars such as Yang [26] also supports that physical exercise can not only directly improve students’ social–emotional competence but also indirectly foster its development by enhancing individuals’ physical and mental health. Specifically, physical exercise strengthens students’ physical fitness, making them more confident and energetic in social interactions [27]. Simultaneously, it serves as an effective method for emotion regulation, helping students alleviate stress and release negative emotions, thereby enabling them to approach social situations with greater composure and positivity [28]. Furthermore, physical exercise cultivates teamwork awareness and communication skills, which are integral components of social–emotional competence. Therefore, encouraging university students to participate actively in physical exercise is of great significance for enhancing their social–emotional competence. In sum, Hypothesis 1 is supported.

Second, the results indicate significant correlations among emotion regulation, physical exercise, and social–emotional competence, with emotion regulation mediating the relationship between physical exercise and social–emotional competence. This suggests that physical exercise can influence emotion regulation, which in turn affects social–emotional competence, a finding consistent with the research of Nelis et al. [29]. According to emotion regulation theory, when individuals face challenges and difficulties during physical exercise, they experience various emotional responses such as anxiety, tension, and excitement. Through effective emotion regulation, individuals can better manage these emotions, thereby maintaining a positive mindset and course of action [30]. To investigate the mediating effect of emotion regulation in greater detail, this study separately examined two distinct strategies: cognitive reappraisal and expressive suppression [31]. The results revealed a significant mediating effect of cognitive reappraisal between physical exercise and social–emotional competence, indicating that cognitive reappraisal is an important mechanistic variable through which physical exercise influences social–emotional competence. In this mediating pathway, the impact of physical exercise on social–emotional competence was more substantial when individuals employed cognitive reappraisal. This positive emotion regulation strategy not only helps individuals exhibit greater confidence, optimism, and empathy in social interactions but also enhances their communication skills and teamwork spirit, thereby improving their social–emotional competence. In contrast, while expressive suppression also demonstrated a certain mediating effect in this study, indicating its role as a significant mechanistic variable, the pathway differs. Here, physical exercise promoted the use of expressive suppression, potentially related to its positive role in improving emotional state and enhancing emotion management capacity [32]. Even when individuals used expressive suppression, physical exercise could, to some extent, mitigate the potential negative impact of this strategy on social–emotional competence. In summary, emotion regulation plays a significant mediating role in the relationship between physical exercise and social–emotional competence. The mediating effect of cognitive reappraisal was more prominent than that of expressive suppression. This finding corroborates related research, as existing studies have confirmed that physical exercise positively influences emotion regulation, enabling individuals to achieve better emotional control [33], thereby enhancing their social–emotional competence in social contexts. Therefore, the identification of emotion regulation as a mediating variable not only reveals the internal mechanism through which physical exercise affects social–emotional competence but also provides a theoretical basis and practical guidance for utilizing physical exercise to enhance university students’ social–emotional competence. Future research should further explore the differential mediating effects of various emotion regulation strategies and investigate how targeted interventions can optimize these effects to more effectively improve students’ social–emotional competence. Thus, Hypothesis 2 is supported.

Third, the study reveals significant correlations among self‐efficacy, physical exercise, and social–emotional competence, with self‐efficacy serving as a mediator in the relationship between physical exercise and social–emotional competence. This indicates that physical exercise can influence self‐efficacy, which subsequently affects social–emotional competence. Grounded in self‐efficacy theory, self‐efficacy refers to the strength of an individual’s conviction in their capability to organize and execute courses of action required to achieve designated types of performances [34]. When engaging in physical exercise, this confidence motivates university students to participate more proactively, strive to overcome fatigue and discomfort during exercise, and consequently achieve better workout outcomes [35]. These improved results, in turn, further enhance their self‐efficacy, establishing a positive feedback loop [36]. The enhancement of self‐efficacy is not confined to physical exercise; it extends to other aspects of students’ lives, such as social interactions and academic pursuits. When students develop higher self‐efficacy through physical exercise, they tend to become more confident and composed in social settings. This enables them to better understand and respond to others’ emotions, engage in effective communication and collaboration, and ultimately enhance their social–emotional competence. Thus, self‐efficacy serves as a crucial link connecting physical exercise and social–emotional competence. Therefore, Hypothesis 3 is confirmed.

Finally, the results demonstrate that emotion regulation and self‐efficacy jointly serve as serial mediators in the relationship between physical exercise and social–emotional competence. This indicates that physical exercise can not only directly influence university students’ social–emotional competence but also exert indirect effects through the intermediary pathways of emotion regulation and self‐efficacy. According to social interaction theory, when individuals can effectively regulate their emotions, they are more likely to experience a sense of accomplishment and satisfaction during physical exercise. These positive emotional experiences, in turn, enhance their self‐confidence and self‐efficacy [37], thereby promoting their performance in social interactions and improving their social–emotional competence. Furthermore, this study incorporated both dimensions of emotion regulation—cognitive reappraisal and expressive suppression—along with self‐efficacy into the theoretical model, thereby revealing the operational mechanisms and pathways among the key variables.

The findings of this study revealed that both serial mediation paths—“physical exercise → cognitive reappraisal → self‐efficacy → social–emotional competence” and “physical exercise → expressive suppression → self‐efficacy → social–emotional competence”—demonstrated significant effects. These results indicate that emotion regulation and self‐efficacy form an interconnected and mutually influencing serial mediation mechanism in the process through which physical exercise promotes university students’ social–emotional competence.

In the pathway “physical exercise → cognitive reappraisal → self‐efficacy → social–emotional competence,” physical exercise prompts students to adopt more positive cognitive reappraisal strategies when confronting challenges and difficulties. This reduces the impact of negative emotions [38], thereby strengthening their self‐confidence—when facing adversities. Such enhanced self‐confidence enables students to engage more proactively in social interactions, better understand and respond to others’ emotions, and ultimately improve their social–emotional competence.

In the pathway “physical exercise → expressive suppression → self‐efficacy → social–emotional competence,” although expressive suppression involves inhibiting the outward expression of emotions, the positive effects of physical exercise may partially mitigate its potential negative impact on social–emotional competence. Physical exercise enhances individuals’ emotional state and improves emotion management capacity [39], enabling students to maintain a certain level of social interactive ability even when employing expressive suppression. At the same time, the self‐efficacy enhanced through physical exercise encourages students to behave more confidently and calmly in social situations, thereby supporting the development of their social–emotional competence.

In summary, the serial mediating effects of emotion regulation and self‐efficacy play an important role in the process by which physical exercise enhances university students’ social–emotional competence. This discovery provides new insights and methodologies for more effectively improving students’ social–emotional capacities and suggests promising directions for future research. Therefore, Hypothesis 4 is confirmed.

## 5. Limitations and Future Research

First, regarding the selection of research variables, this study did not comprehensively cover all important factors influencing university students’ physical exercise and social–emotional competence, such as social support, psychological resilience, and personality traits. Future studies should, therefore, integrate both external environmental factors and intrinsic individual characteristics and conduct comprehensive analyses building upon existing research to provide scientific guidance for developing targeted intervention strategies. Second, in terms of sampling, this study only investigated 572 students from Jiangxi Science and Technology Normal University, resulting in a relatively homogeneous sample source that may limit the representativeness and statistical power of the findings. Subsequent research should employ cluster random sampling methods across multiple regions in China, expanding both the geographic coverage and sample size to enhance the generalizability and explanatory power of the results. Third, methodologically, this cross‐sectional design preliminarily revealed surface‐level relationships among variables but cannot establish causal inferences. Future research should prioritize longitudinal intervention experiments or integrate cross‐sectional and longitudinal designs through multiwave data collection to more systematically investigate the dynamic relationships and causal pathways between physical exercise and social–emotional competence, thereby providing more solid evidence for theoretical development and empirical support in this field. Fourth, this study found that expressive suppression, a dimension of emotion regulation, and self‐efficacy play a partial positive role between physical exercise and social–emotional competence. While this finding shows both consistencies and inconsistencies with previous research, preventing definitive conclusions, it suggests that additional mediating variables and regional characteristic differences may influence the relationship between physical exercise and social–emotional competence. Future studies should continue to investigate other potential variables in this mechanism. Additionally, the authors will also consider including multiple measurement tools in future research.

## 6. Conclusions and Recommendations

The mediation model constructed in this study partially elucidates the mechanism through which physical exercise predicts social–emotional competence among students at Jiangxi Science and Technology Normal University. Increased engagement in physical exercise can directly predict higher levels of social–emotional competence, and it can also exert an indirect predictive effect through the mediating roles of either emotion regulation or self‐efficacy alone. Furthermore, it predicts university students’ social–emotional competence via the serial mediation pathway of emotion regulation and self‐efficacy.

Based on these findings, we propose the following three recommendations to promote the development of university students’ social–emotional competence. First, although the overall physical activity level of the students in this study was moderate, more than half remained at a low level. Therefore, it is recommended that the student population maintain regular physical exercise over the long term. Exercise load and intensity should be adjusted appropriately based on individual physiological conditions and health needs to ensure that sustained and scientific physical activity effectively achieves the intended health benefits.

Second, within the context of the national fitness campaign, it is recommended to encourage university students to actively participate in social sports activities, team‐based exercises, and volunteer service projects by providing diverse social platforms and activity opportunities. This will help enhance their interpersonal skills and facilitate the construction of social networks. Simultaneously, efforts should be made to actively promote an inclusive culture and a supportive environment, emphasizing respect and care in social interactions, thereby fostering the development of self‐confidence and emotional stability. Such measures will lay a solid foundation for students’ social integration and the cultivation of emotional competence. At the institutional level, universities should attach great importance to the cultivation of students’ positive emotions and integrate this objective into curriculum reform and the teaching system. Classroom instruction should go beyond knowledge transmission to include guidance on emotional awareness and management skills. Through diverse channels such as group activities, students should be provided with opportunities to experience and express positive emotions, thereby holistically enhancing their psychological well‐being and social–emotional competence. At the family level, parents are encouraged to actively participate in their children’s physical exercise and jointly develop habits of regular physical activity. Sports‐related interactions can strengthen parent–child communication and help build a family atmosphere characterized by understanding and support, thereby enhancing children’s self‐efficacy. Through encouragement and positive feedback, parents can also guide their children to master emotion regulation techniques, strengthen their self‐control capacity and positive efficacy beliefs, and gradually help them develop sound social–emotional concepts and behavioral patterns. This will lay a robust foundation for the establishment of their social values.

Third, in the practice of emotion regulation, it is recommended to scientifically select appropriate strategies based on the neural mechanisms and operational processes of emotion regulation. Through systematic training, individuals can enhance their capacity for emotional modulation, thereby promoting the alignment of psychological and behavioral responses. This process not only contributes to the improvement of self‐efficacy but also holds positive significance for the comprehensive enhancement of social–emotional competence.

## Author Contributions

**Longan Cao**: conceptualization, data curation, formal analysis, methodology, software, writing – original draft. **Jianhui Liu**: investigation, validation, writing – review and editing. **Lei Wang and Jun Lang**: investigation, visualization, writing – review and editing. **Zhengban Ran**: conceptualization, project administration, supervision, writing – review and editing.

## Acknowledgments

We sincerely appreciate all participants for their valuable contributions to this study, as well as acknowledge the assistance provided by team members in data collection and preprocessing. Meanwhile, during the process of writing this work, the authors utilized DeepSeek and ChatGPT to enhance the grammatical correctness of the article. Therefore, we would like to express our gratitude to the artificial intelligence software for optimizing the grammar of this text.

## Funding

This study was funded by the Chinese National Social Science Foundation (Project Name: Research on the Disciplinary Development Logic of Sports Science in China in the New Era, Project Number 21BTY019). This study was supported by the National Natural Science Foundation of China (Title: Research on Modeling Human Emotional Physiological Response System and Its Application, Grant 61472330).

## Disclosure

After using DeepSeek and ChatGPT, the authors reviewed and edited the content as needed and takes full responsibility for the content of the publication.

## Ethics Statement

The studies involving human participants were reviewed and approved by the Ethics Committee at South West University (Approval Number 20250618B2). According to the national laws and institutional regulations, the collection of survey data has obtained written informed consent from all participants. Upon completion of the survey, the research team provided each participant with a small token of appreciation (a commemorative gift valued at 2 RMB) in recognition of their time and contribution.

## Conflicts of Interest

The authors declare no conflicts of interest.

## Supporting Information

Additional supporting information can be found online in the Supporting Information section.

## Supporting information


**Supporting Information** The supporting information contains the research data of this study, which can be used as a reference for readers.

## Data Availability

The data that support the findings of this study are available in the supporting information of this article.
